# Clinical characteristics of stress cardiomyopathy in patients with acute poisoning

**DOI:** 10.1038/s41598-017-18478-5

**Published:** 2018-01-09

**Authors:** Ung Jeon, Samel Park, SangHo Park, Eun-young Lee, Hyo-Wook Gil

**Affiliations:** Department of Internal medicine, Soonchunhyang University Cheonan Hospital, Cheonan, Republic of Korea

## Abstract

Patients who attempt intentional suicide suffer from physical or emotional stress. This situation might be an important factor that causes takotsubo cardiomyopathy. We retrospectively investigated the clinical features of Takotsubo cardiomyopathy in patients with acute poisoning. This study included patients who were admitted from January 2010 to December 2015 because of intentional poisoning by ingestion. Among these patients, we selectively collected data of patients who underwent an echocardiogram. We divided the patients into three groups according to the echocardiogram; the non-cardiomyopathy group, the global hypokinesia group, and the takotsubo cardiomyopathy group. One hundred forty-seven patients were analyzed in this study. One hundred thirty-one patients had normal cardiac function without regional wall motion abnormality. Global hypokinesia was observed in five patients. The overall incidence of takotsubo cardiomyopathy was 7.5% (11/147). Levels of cardiac enzymes including CK-MB, Troponin T, a marker of cardiac muscle ischemia, were higher in the global hypokinesia group and the takotsubo cardiomyopathy group compared with the non-cardiomyopathy group. The most commonly consumed poison was organophosphate in the takotsubo cardiomyopathy group. In conclusion, takotsubo cardiomyopathy may be one of the cardiac complications in patients who attempt suicide by consuming a poison.

## Introduction

In 2014, 2,165,142 human exposures were reported in USA^1^. The mortality rate was less than 5%, but the mortality rate or complications could be different according to the poison^2,3^. Although systemic symptoms are different according to the poison, cardiovascular complication is important in acute poisoning. Poisons can reduce cardiac contractility, resulting in a decrease in the cardiac ejection fraction and cardiac output, hypotension, and development of congestive heart failure. A cardiogenic event generally occurs as a result of the direct effects of poisons on contractility or inotropy of the heart. However, some reports have shown that takotsubo cardiomyopathy, also known as transient left ventricular ballooning syndrome or stress cardiomyopathy, could be one of the causes of cardiac complication in patients with acute poisoning^4–7^. Takotsubo cardiomyopathy is characterized by transient, reversible ventricular dysfunction with normal coronary arteries^8^. Acute physical or emotional distress is thought to play a role in the development of stress cardiomyopathy through spike-like sympathetic stimulation^9^. Patients who attempt intentional suicide suffer from physical or emotional stress. This situation might be an important factor that causes takotsubo cardiomyopathy. Although there are some published case reports of stress-induced cardiomyopathy in patients with acute poisoning^5–7,10^, the incidence or clinical course of takotsubo cardiomyopathy in these patients has not been evaluated. Therefore, we retrospectively investigated the clinical features of Takotsubo cardiomyopathy in patients with acute poisoning.

## Materials and Methods

### Study population

This study included patients who were admitted to the Soonchunhyang University Cheonan Hospital from January 2010 to December 2015 because of intentional poisoning by ingestion. Among these patients, we selectively collected data of patients who underwent an echocardiogram. The present study was approved by Soonchunghyang Cheonan Hospital’s Institutional Review Board. The requirement for obtaining informed consent was waived because of the retrospective design of this study. This study was conducted in accordance with the principles of the Declaration of Helsinki. When patients had unexplained hypotension, unexplained dyspnea, elevation of cardiac enzyme levels or electrocardiogram abnormality, we consulted a cardiologist who decided whether to perform a transthoracic echocardiogram. Transthoracic echocardiographic examinations were performed by experienced sonographers by using a 2.5 MHz transducer attached to a commercially available Doppler echocardiogram and then, they were interpreted by expert cardiologists.

Echocardiographic images included parasternal long and short axis views, and apical 4, 3, and 2 chamber views, and if it was difficult to obtain these views, we obtained a 4 chamber and short axis view through subcostal views, according to the guidelines of the American Society of Echocardiography. Measures of ejection fraction (EF) represent the cardiac function of the assessed patients. EF was measured using the modified Simpson’s technique. Global dysfunction refers to an overall decrease in left ventricular (LV) wall motion, and regional wall motion abnormality indicates a partial decrease in some segments of the LV wall. Exclusion criteria were age less than 18 years and patients with inhalation poisoning.

## Study variables and Definitions

We divided the patients into three groups according to the echocardiogram; the non-cardiomyopathy group, the global hypokinesia group, and the takotsubo cardiomyopathy group. The non-cardiomyopathy group included patients with normal left ventricular function without regional wall motion abnormality. The global hypokinesia group included patients with a global decrease in left ventricular wall motion. The Takotsubo cardiomyopathy group included patients with localized hypokinesia, akinesia, dyskinesia of the left ventricular segments with or without apical involvement; the regional wall motion abnormalities extended beyond a single epicardial vascular distribution, which had recovered on follow-up echocardiogram. We documented patient age, sex, active toxic compounds, and laboratory findings. We also investigated acute kidney injury (AKI), use of mechanical ventilation, vasopressor or atropine usage, and mortality. AKI was defined as a 2-fold increase from baseline creatinine.

## Statistical analysis

Continuous variables are presented as the mean ± SD, with or without the median value and range, and categorical variables are presented as the frequency (the number of cases and percentage). The differences among groups were analyzed using Student’s t-test or the Mann-Whitney U test for continuous variables and the chi-square test or Fisher’s exact test for categorical variables. To analyze the differences among >3 groups, we used the Kruskal-Wallis test. Statistical analyses were performed using SPSS software, version 14.0 (SPSS, Chicago, IL, USA). P values less than 0.05 were considered statistically significant.

## Results

**Figure 1:**
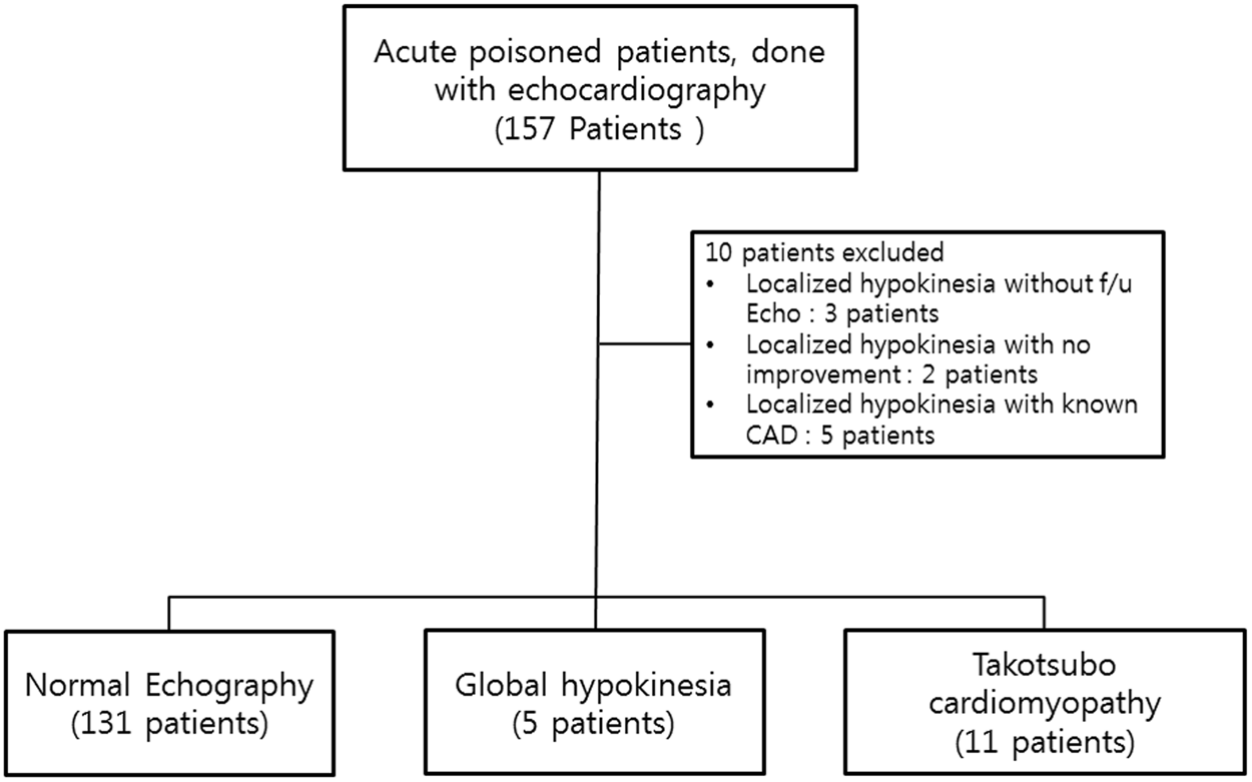
Flow diagram of patient selection.

In this period, a total of 2866 patients were admitted to our hospital due to acute poisoning. Echocardiogram was performed in 157 patients. A total of 10 patients were excluded due to the following reasons: localized hypokinesia without follow-up echocardiogram (3 subjects), localized hypokinesia without improvement on follow-up echocardiogram (2 subjects), and localized hypokinesia with known coronary artery disease (5 subjects) (Fig. 1). Finally, 147 patients were analyzed in this study. One hundred thirty-one patients had normal cardiac function without RWMA. Global hypokinesia was observed in five patients. The overall incidence of takotsubo cardiomyopathy was 7.5% (11/147).

The baseline characteristics are shown in Table 1. There was no difference in sex among these three groups. According to the poison, the incidence of takotsubo cardiomyopathy was not significantly different. Vasopressin and mechanical ventilation were used more frequently in the takotsubo cardiomyopathy group compared with the others groups, although there were no differences in atropine use and incidence of AKI among the three groups.

**Table 1:** Characteristics of patients according to the pattern of cardiomyopathy.

	Non-cardiomyopathy (n = 131)	Global hypokinesia (n = 5)	Takotsubo cardiomyopathy (n = 11)	p-value
Sex (M/F)	73/58	4/1	5/6	0.455
Age	67.39 ± 14.72	69.00 ± 10.00	63.45 ± 14.03	0.425
Underlying disease				
DM	21(16.0%)	1(20.0%)	1(9.1%)	0.741
Hypertension	62(47.3%)	2(40.0%)	5(45.5%)	1.000
Poisoning				0.547
Pesticide				
Herbicide				
Glyphosate	17	1	1	
Glufosinate	24	1	4	
Glyphosate + Glufosinate	3	0	0	
Paraquat	12	1	0	
Other herbicides	6	0	0	
Insecticide				
Organophosphate	8	1	4	
Pyrethroid	4	0	0	
Organophosphate + other insecticide	7	0	1	
Carbamate	4	0	0	
Other insecticides	5	0	0	
Fungicide	1	0	1	
Medical drug	27	1	0	
pesticide + medical drug	3	0	0	
others	7	0	0	
Unknown	3	0	0	
Medical complication				
Mechanical ventilation	62 (47.3%)	2 (40.0%)	10 (90.9%)	0.012
Acute kidney injury	52 (39.7%)	4 (80.0%)	7 (63.6%)	0.065
Medication during hospitalization				
Vasopressor	52 (39.7%)	3 (60.0%)	9 (81.8%)	0.011
Atropine	23 (17.6%)	1 (20.0%)	4 (36.4%)	0.259
Mortality	14 (10.7%)	0 (0.0%)	0 (0.0%)	0.772

Comparisons of echocardiographic and laboratory findings among these three groups are shown in Table 2. EF was lowest in the takotsubo cardiomyopathy group compared with the other groups. The level of peak Troponin T, a marker of cardiac muscle ischemia, was higher in the global hypokinesia and takotsubo cardiomyopathy groups compared with the non-cardiomyopathy group.

**Table 2:** Comparison of laboratory among the three groups.

	Non-cardiomyopathy (n = 131)	Global hypokinesia (n = 5)	Takotsubo cardiomyopathy (n = 11)	p-value
Ejection fraction	64.98 ± 4.91	47.20 ± 5.59	34.27 ± 6.20	<***0***.***001***
Heart rate	87.42 ± 20.69	77.00 ± 16.81	104.55 ± 16.35	***0***.***008***
White blood cell count	13732 ± 5697	13164 ± 9745	15302 ± 6810	0.657
Hospital days	16.11 ± 12.86	11.80 ± 5.63	24.55 ± 11.10	***0***.***023***
Hemoglobin (g/dL)	13.84 ± 1.86	12.28 ± 3.85	13.27 ± 1.60	0.123
Platelet	252.92 ± 101.05	175.00 ± 30.14	243.09 ± 48.20	***0***.***033***
Albumin (mg/dL)	4.08 ± 0.57	3.92 ± 0.59	4.10 ± 0.62	0.806
Glucose (mg/dl)	162.76 ± 64.86	154.00 ± 53.49	165.27 ± 62.37	0.982
AST (IU/L)	43.11 ± 53.61	38.20 ± 17.34	47.64 ± 44.12	0.575
ALT (IU/L)	29.05 ± 53.75	19.40 ± 7.89	26.64 ± 21.73	0.951
LDH (IU/L)	314.98 ± 152.76	445.80 ± 290.20	352.09 ± 194.65	0.550
Urea nitrogen (mg/dL)	18.00 ± 9.16	22.60 ± 6.20	15.15 ± 5.03	0.101
Creatinine (mg/dL)	1.02 ± 0.60	1.58 ± 0.79	0.76 ± 0.17	***0***.***033***
Calcium (mg/dL)	8.86 ± 1.29	8.24 ± 0.17	8.47 ± 0.91	***0***.***046***
Phosphorus (mg/dL)	3.63 ± 1.54	4.06 ± 2.33	3.22 ± 1.12	0.564
Uric acid	5.40 ± 2.15	5.84 ± 2.72	3.72 ± 1.28	***0***.***018***
Sodium (mEq/L)	139.87 ± 4.48	142.00 ± 5.83	141.00 ± 3.35	0.642
Potassium (mEq/L)	3.97 ± 0.73	4.36 ± 1.02	3.86 ± 1.00	0.525
Chloride (mEq/L)	102.08 ± 5.33	106.60 ± 6.95	104.64 ± 4.76	0.108
pH	7.35 ± 0.11	7.32 ± 0.14	7.36 ± 0.13	0.934
PaCO2 (mmHg)	35.93 ± 9.88	31.80 ± 5.81	39.78 ± 14.62	0.475
PaO2 (mmHg)	85.31 ± 41.40	83.00 ± 30.24	89.97 ± 66.67	0.948
HCO3 (mEq/L)	20.11 ± 5.31	16.88 ± 5.31	21.82 ± 4.80	0.266
Base excess	−5.479 ± 6.50	−9.24 ± 7.25	−3.55 ± 5.60	0.386
Creatine kinase	822.56 ± 4970.86	695.80 ± 870.92	122.82 ± 65.47	0.203
Peak CK-MB*	55.54 ± 82.46	68.60 ± 66.05	63.45 ± 44.55	0.123
Peak troponin T*	0.1197 ± 0.4172	0.1646 ± 0.2635	0.2218 ± 0.1694	***0***.***001***
Peak NT-proBNP*	1265.44 ± 1937.08	371.40 ± 41.71	9728.19 ± 10061.51	***0***.***003***
CRP	20.80 ± 56.31	60.16 ± 100.43	10.85 ± 21.65	0.374

AST, aspartate transaminase; ALT, alanine transaminase; LDH, lactate dehydrogenase; CK-MB, creatine kinase-MB; NT-proBNP, N-terminal prohormone of brain natriuretic peptide.

**Figure 2:**
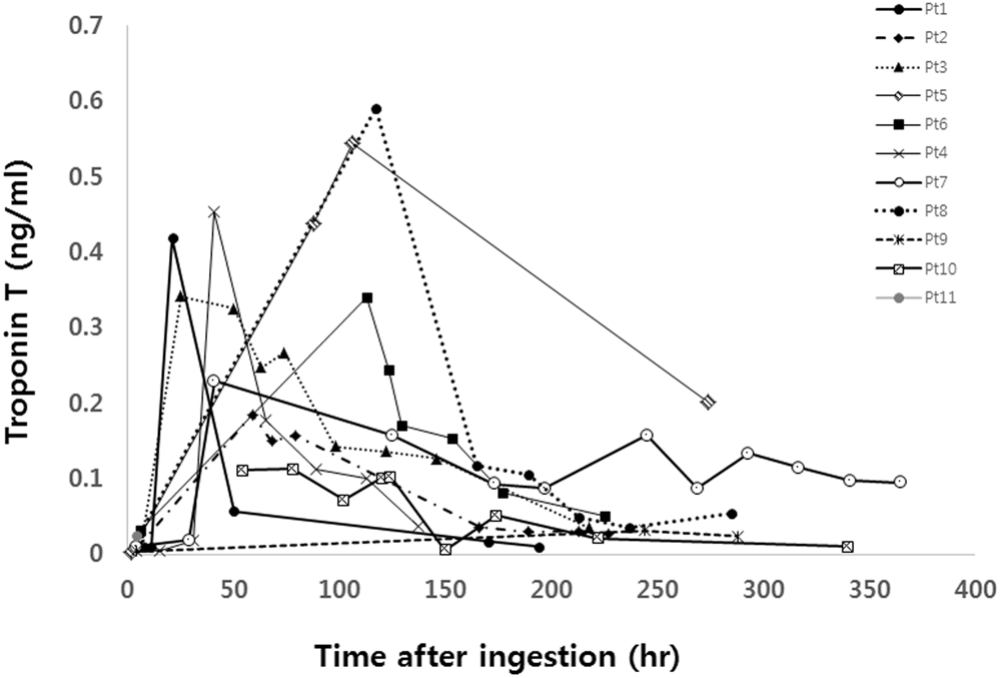
Serial change in Troponin T versus time lag after ingestion in the takotsubo cardiomyopathy group.

Individual characteristics of patients with takotsubo cardiomyopathy are shown in Table 3. The most commonly consumed poison was organophosphate and the second most commonly consumed poison was glufosinate. In 5 patients with organophosphate intoxication, there were 3 cases of the mid-ventricular type and 2 cases of the basal type although the apical type was more common in glufosinate intoxicated patients. Figure 2 shows the individual pattern of troponin T in takotsubo cardiomyopathy. Initial troponin T until 24 hr after ingestion was within normal limits in patients.

**Table 3:** Individual characteristics of patients with takotsubo cardiomyopathy.

Age/sex	Poisoning drug	Initial echocardiograpic findings	Initial EF (%)	ECG findings	Peak Pro-BNP (pg/ml)	Peak CK-MB (U/L)	Peak Troponin T (ng/ml)	F/U EF (%)	F/U days of echo	Coronary evaluation (CAG or cardiac angio CT)
50/F	Organophospate	Mid ventricular type	40	T wave inversion	4113	97	0.342	58	8	NA
79/M	Organophospate	Mid ventricular type	31	Deep T wave inversion	745.6	49	0.230	64	8	No significant coronary stenosis
54/F	Organophospate	Basal type	35	No ST-T change	13061	35	0.086	51	6	NA
51/F	Organophospate	Mid ventricular type	26	ST depression	8678	79	0.118	57	7	NA
54/F	Glyphosate	Apical type	25	T wave inversion	180.5	62	0.419	58	7	No significant coronary stenosis
80/M	Glufosinate	Apical type	38	Deep T wave inversion	12749	21	0.113	65	60	NA
43/F	Glufosinate	Basal type	45	ST depression	472.8	44	0.454	62	8	No significant coronary stenosis
71/F	Glufosinate	Apical type	35	ST elevation Deep T wave inversion	18877	55	0.043	66	18	No significant coronary stenosis
71/M	Glufosinate	Apical type	30	Deep T wave inversion	-	36	0.185	60	60	No significant coronary stenosis
53/M	Organophospate	Basal type	32	No ST-T change	32193	179	0.544	71	20	NA
81/M	Organophospate + pyrethroid	Apical type	40	ST elevation Deep T wave inversion	6212	60	0.341	55	8	NA

EF: ejection fraction, ECG: electrocardiography, F/U: follow up, CAG: coronay angiography, CT: computer tomography, NA: not available.

In our study, 14 fatal cases were observed in the type I group. There was no death in the global hypokinesia group and the takotsubo cardiomyopathy group. The causes of deaths were as follows: paraquat (paraquat induced lung injury) in 2 cases; Organophosphate in 4 cases (sudden cardiac arrest in one case, pneumonia in one case, and multiorgan failure in two cases); pyrethroid (pneumonia) in one case; medical drugs in 3 cases (pneumonia in 2 cases, neuroleptic malignant syndrome in one case); glyphosate (pneumonia, mutiorgan failure) in 2 cases; glyphosate + glufosinate (sudden cardiac death) in one case; and other herbicide (methemoglobinemia) in one case.

## Discussion

Our study suggested that the incidence of takotsubo cardiomyopathy was approximately 7.4% among patients with acute poisoning who had undergone echocardiography. Among the total 2866 patients, the estimated incidence of takotsubo cardiomyopathy was approximately 0.4%.

A previous study has shown that patients with takotsubo cardiomyopathy had a higher prevalence of neurological or psychiatric disorders than did those with an acute coronary syndrome^11^. Suicide attempt might be a very emotionally and physically stressful condition^12^. Emotional stress might be one of the important risk factors in the development of stress cardiomyopathy^13^. Deshmukh A *et al*. showed that Takotsubo cardiomyopathy was diagnosed in approximately 0.02% of all hospitalizations in the United States, mostly in elderly women with a history of smoking, alcohol abuse, anxiety state, and hyperlipidemia^14^. Our study suggested that the incidence of takotsubo cardiomyopathy was higher in patients with acute poisoning than in the general admitted patients^15,16^.

Although the pathogenesis of takotsubo cardiomyopathy is not well understood, a number of features suggest that it may be caused by diffuse catecholamine-induced microvascular spasm or dysfunction, resulting in myocardial stunning. Also, increasing acetylcholine levels in the coronary artery would cause stress-induced cardiomyopathy. Our study showed that organophosphate insecticide and glufosinate herbicide were the main poisons in the stress cardiomyopathy group. Organophosphate insecticide inhibits acetylcholinesterase, which results in accumulation of acetylcholine at the autonomic and some central synapses and at the autonomic postganglionic and neuromuscular junctions^17^. One previous study has shown that severe acute dichlorvos poisoning is associated with reversible myocardial dysfunction, possibly through an increase in catecholamine levels^18^. Organophosphorus poisoning might be related with a high catecholamine level. These findings suggest that the clinician should be aware that stress cardiomyopathy could be related to hypotension in organophosphorus insecticide poisoning. These findings suggest that the clinician should be aware that stress cardiomyopathy could be one complication in organophosphorus insecticide poisoning.

Our study showed the takotsubo cardiomyopathy type is different compared with the previous report^11^. Tempin C *et al*. showed that apical takotsubo cardiomyopathy was identified in 81.7% of patients, whereas the midventricular form was found in 14.6% of patients, and basal and focal forms were diagnosed in 2.2% and 1.5% of patients, respectively^11^. Although the characteristics of different types are not fully explained, some studies have suggested that the location or amount of adrenoreceptor according to age and sex may lead to a different ballooning pattern of takotsubo cardiomyopathy. Our study showed that apical takotsubo cardiomyopathy was identified in 5 of 11 patients (45.4%), and midventricular form and basal form were individually found in 27.3% of patients. Interestingly, in 5 organophosphate poisoned patients, midventricular form was found in 3 patients and basal form was observed in 2 patients. Further study should reveal whether adrenoreceptor location could be associated with the incidence of takotsubo cardiomyopathy among organophosphate poisoned patients.

Glufosinate is a structural analogue of glutamic acid, a typical excitatory amino acid in the central nervous system (CNS), the main target of acute glufosinate poisoning, although the underlying cellular and molecular mechanisms of this action are not understood clearly^19^. Glufosinate is thought to inhibit glutamine synthetase and glutamine decarboxylase, resulting in decreased glutamic acid levels and CNS symptoms (drowsiness, memory impairment, and seizures). A majority of this class of herbicides contain an anionic surfactant that increases blood permeability, resulting in decreasing circulatory blood volume, cardiac function, and resistance of systemic peripheral vessels^20,21^. One case report showed that Takotsubo cardiomyopathy was a delayed complication with a herbicide containing glufosinate ammonium in a suicide attempt^7^. Surfactant volume might be an important factor in patients with surfactant-containing herbicide poisoning^21–23^. Further study should focus on the relationship between stress cardiomyopathy and surfactant volume.

Our study has some limitations. First, our study used a retrospective design. Further prospective study should reveal the incidence and type of Tachotsubo cardiomyopathy in acute poisoning patients. Second, selection bias could have affected the interpretation of the results because our study only included those patients who had undergone echocardiography. Third, poisons could differ with other centers. Our center is a specialized pesticide poisoning treatment center. Our study population could be more severely affected compared with those in other studies. Fourth, we included patients without performing full coronary disease evaluation according to the Mayo clinic diagnostic criteria. The Mayo clinic’s Takotsubo definition should satisfy both the absence of significant stenosis on coronary angiography with transient RWMA. Although the mayo clinic criteria have been used, Takotsubo cardiomyopathy does not have a universally accepted diagnostic definition^24^. In our study, coronary evaluation was performed in only 5 of 11 patients included in TCM. Blind reading of initial echocardiogram by two experts cardiologist showed a suspicious RWMA pattern of takotsubo cardiomyopathy and follow-up echocardiography revealed complete recovery of RWMA. Also in clinical course, the delayed elevation pattern and moderate level of troponin T might support the diagnosis of Takostubo cardiomyopathy. But, we believe that the diagnosis of takotsubo cardiomyopathy is not doubtful because of these reasons. initial and follow-up echocardiography showed a reversed recovery and typical pattern, and cardiac enzyme levels supported the findings.

In conclusion, takotsubo cardiomyopathy could be considered as one of the cardiac complications in patients who attempt suicide by consuming a poison.
